# Time to trimethoprim/sulfamethoxazole initiation among patients with rheumatic disease complicated by *Pneumocystis jirovecii* pneumonia: impact on 90-day mortality

**DOI:** 10.1186/s12879-022-07940-z

**Published:** 2022-12-27

**Authors:** Siyang Song, Yang Zhang, Jie Yu, Cuiying Xie, Yi Chen, Xingyu Zhang

**Affiliations:** Department of Emergency, Shanghai Jiaotong University School of Medicine Affiliated Renji Hospital, 2000 Jiangyue Road, Minhang District, Shanghai, China

**Keywords:** *Pneumocystis jirovecii* pneumonia, Rheumatic disease, Trimethoprim, Sulfamethoxazole, Systemic lupus erythematosus, Inflammatory myopathy

## Abstract

**Background:**

*Pneumocystis jirovecii* pneumonia (PJP) is a life-threatening disease with increasing prevalence in patients with rheumatic disease. Trimethoprim/sulfamethoxazole (TMP/SMX) is an effective treatment for patients with rheumatic disease hospitalized for PJP. This study aimed to describe the 90-day mortality of patients with rheumatic disease complicated by PJP and investigate whether the administration of TMP/SMX after 7 days from initial symptoms correlates with 90-day mortality.

**Methods:**

We enrolled consecutive patients with rheumatic disease complicated with PJP in our center from August 2018 to August 2021. The participants were classified into two groups according to when TMP/SMX was initiated: early (within the first 7 days) and late (after 7 days). The primary outcome was 90-day PJP-related mortality. Multivariate cox regression and Kaplan–Meier survival analyses were conducted to identify the risk factors for mortality and examine differences in survival between early and late use of TMP/SMX.

**Results:**

Thirty-seven patients with rheumatic disease (median age 50.1 years, 24.3% male) complicated by PJP were enrolled in our study, and 15 (40.5%) patients died at or before 90 days of follow-up. The most common comorbidity was systemic lupus erythematosus (14, 37.8%), followed by inflammatory myopathy (11, 27.9%). Patients in the early group were less likely to require mechanical ventilation (8/27, 29.6% vs. 9/10, 90.0%, P = 0.002), lower doses glucocorticoids (43.2 mg/d vs. 72.2 mg/d, P = 0.039) and had lower mortality (7/27, 25.9% vs. 8/10, 80.0%, P = 0.006) than those in the late group. In the Kaplan–Meier analysis, the survivor probability of the early group was notably higher than that of the late group (P = 0.007). Multivariate cox regression analysis showed that initiation of TMP/SMX after 7 days from admission (hazard ratio [HR]: 5.9, 95% confidence interval [CI]: 1.1–30.4; P = 0.034) and a higher level of lactate dehydrogenase (LDH; HR: 6.0, 95% CI: 1.1–31.8; P = 0.035) were associated with 90-day mortality in patients with rheumatic disease complicated by PJP.

**Conclusion:**

Patients with rheumatic disease complicated by PJP had poor prognoses, with mortality rates as high as 40.5%. TMP/SMX initiation after 7 days from initial symptoms and a higher level of serum LDH were significantly associated with increased 90-day mortality.

## Background

*Pneumocystis jirovecii* pneumonia (PJP) is caused by an opportunist fungus that may cause severe or even fatal outcomes in immunocompromised patients, with an incidence approaching 400,000 cases per year globally [1]. Although PJP was initially identified during the acquired immune deficiency syndrome epidemic, its mortality has declined significantly over the past decades due to the use of highly active antiretroviral therapy [2]. In contrast, it has emerged as a significant cause of a typical pneumonia among non-human immunodeficiency virus (HIV) immunocompromised patients who received steroids and cytotoxic drugs [3], the principal therapeutic agents for many rheumatic diseases, which include systemic lupus erythematosus (SLE), inflammatory myopathy (IM), Sjogren’s syndrome, Adult-onset Still's disease, Anti-synthetase syndrome, and rheumatoid arthritis.

The precise incidence of PJP in rheumatic diseases is unknown, but a previous study depicted a substantially higher prevalence in patients with SLE, inflammatory myositis, and Wegener’s granulomatosis [4]. More importantly, PJP in patients with rheumatic diseases and non-HIV tends to manifest more severe symptoms and worse prognoses, paralleled by higher oxygen requirements and the need for mechanical ventilation [5–7]. Effective treatments for PJP in rheumatic diseases are therefore of paramount clinical importance.

Trimethoprim/sulfamethoxazole (TMP/SMX) has been considered the first-line therapy for treating PJP, according to national and international guidelines [8]. However, in clinical practice, the potential harm caused by TMP/SMX administration for patients with rheumatic diseases is also significant, such as drug allergy, renal dysfunction, and thrombocytopenia [9].

Extensive ground-glass opacity is the most typical radiographic finding in PJP [10] on high-resolution computed tomography (CT), which resembles interstitial lung lesions of patients with rheumatic diseases. Therefore, no delay in diagnosing PJP and prescribing TMP/SMX can be challenging due to the nonspecific nature of clinical and radiographic findings, as well as the concerns about adverse effects of the drugs. Recently, it has been shown that prophylactic TMP/SMX dramatically reduces the incidence of PJP in patients with rheumatic diseases who received prolonged, high-dose steroid treatment [11]. However, it is uncertain whether early TMP/SMX administration can improve outcomes in patients with rheumatic disease with definite PJP diagnoses.

We performed this study to describe the risk factors associated with mortality in patients with rheumatic disease complicated by PJP and further assess whether initiating treatment with TMP/SMX within 7 days is independently associated with better clinical outcomes, such as lower rates of mortality and the need for mechanical ventilation, than initiating TMP/SMX after 7 days from initial clinical symptoms.

## Methods

### Study population

This was an observational retrospective study, and we retrospectively reviewed the medical records of patients with rheumatic diseases in our center with discharge diagnoses of PJP in Shanghai Jiao Tong University of Medicine affiliated with Renji Hospital from August 2018 to August 2021. Ethical approval was obtained from Shanghai Jiao Tong University of Medicine, affiliated with Renji Hospital. Per this ethical approval, informed consent was waived by the ethical review committee due to the retrospective and observational nature of the study.

The diagnosis of PJP were made according to clinical manifestations, imaging examinations, and microbiological test results. The criteria were as follows: (1) compatible clinical symptoms including fever, cough, sputum, and dyspnea; (2) radiological findings compatible with PJP such as uni- or bilateral ground-glass opacity or patchy consolidation; and (3) microbiologic finding including conventional or immunofluorescence staining and molecular diagnosis by metagenomics next-generation sequencing (NGS) via bronchoalveolar lavage fluid (BALF) and blood samples. In our study, PJP was identified by NGS of BALF in 25 of the 37 patients, and by NGS of blood in 11 patients, and one patient was diagnosed by conventional microscopic staining of sputum specimens. We excluded patients with any of the following criteria: (1) insufficient data; (2) those receiving TMP/SMX prophylaxis. All cases were reviewed and the diagnosis of PJP in all patients was confirmed by physicians, radiologists, and microbiologists.

### Data collection and definition

All data were obtained from the patients’ electronic and physical medical records, and two trained observers collected anonymous patient data using a pre‑specified case report form. Patients’ information was collected, including demographics, types and history of rheumatic diseases, types of immunosuppressive agents, time from initial symptom to TMP/SMX use, comorbidities, laboratory results on admission, and clinical outcomes.

The cut-off of 7 days from initial symptom to TMP/SMX use was selected based on real-world practice. The dosage of TMP/SMX used for treatment of active PJP is 15–20 mg/kg TMP and 75–100 mg/kg SMX per day [12].

The laboratory data were collected using the worst values recorded within the first 24 h of admission. The cut-off of lactate dehydrogenase (LDH) level was defined as 500 U/L based on published literature [13, 14].

CMV viremia was defined as plasma CMV viral load exceeding 400 copies per mL. CMV infection was defined as evidence of CMV replication regardless of symptoms. CMV disease was defined as evidence of CMV infection and attributable symptoms including viral syndrome and tissue invasive disease [15].

The primary outcome of the study was 90-day PJP-related mortality. We obtained the 90-day outcome of the patient by referring to the patient's medical records of scheduled visits or re-hospitalization records after discharge. If there was no clear medical records, we contacted the patient’s family members by telephone calls to confirm the patient’s 90-day outcome.

### Statistical analysis

Continuous variables are presented as means and standard deviations, whereas categorical variables are presented as frequencies and percentages. For the univariate analysis, categorical variables were compared using the Chi‑square or Fisher’s exact tests. The Mann–Whitney U test was used for analyzing continuous variables as they were non-normally distributed. Uni- and multivariate cox regression analyses were conducted to identify the risk factors for 90-day PJP-related mortality. The variables entered into multivariate analysis were chosen from the factors with P < 0.02 in the univariate analysis. Point estimates are presented as hazard ratios (HRs) with 95% confidence intervals (CIs). The association between TMP/SMX administration within 7 days and mortality was tested by the log-rank test using Kaplan–Meier survival curves. All analyses were performed using SPSS version 23.0.0.0 (SPSS, IBM, USA). P-values < 0.05 were considered statistically significant.

## Results

Data of 46 patients with rheumatic disease who met the inclusion criteria were collected between August 2018 and August 2021. Finally, 37 patients were enrolled, 5 were excluded due to severe data loss, and 4 were excluded because of receiving TMP/SMX prophylaxis.

We divided the patients into the early and late groups based on the time to TMP/SMX initiation whether more than 7 days after administration, respectively. Baseline characteristics and clinical outcomes between the two groups are shown in Table 1. The median age of patients with rheumatic disease with PJP was 50.1 years old, and 75.7% were female. And the 90-day PJP-related mortality of patients with rheumatic disease was 40.5%. There were 27 (73.0%) patients who treated with TMP/SMX within 7 days and 10 (27.0%) patients who treated with TMP/SMX after 7 days. There was no significant difference in demographic characteristics, underlying rheumatic diseases, comorbidities, laboratory data and treatments before admission between the two groups. The primary comorbidity was SLE (14 patients, 37.8%), followed by IM (11 patients, 27.9%). Cyclophosphamide was used in 24.3% of patients, and 21.6% received more than two immunosuppressive agents. There were 43.2% patients had CMV viremia. All PJP patients combined with the rheumatic disease received glucocorticoids therapy. Nevertheless, patients treated with TMP/SMX after 7 days from initial symptoms more likely required mechanical ventilation (90.0% vs. 29.6%, P = 0.002), higher doses glucocorticoids (72.2 mg/d vs. 43.2 mg/d, P = 0.039) and had higher 90-day mortality rate (80.0% vs. 25.9%, P = 0.006) than those treated with TMP/SMX within 7 days.

**Table 1:** Baseline characteristic and clinical outcome of patients with rheumatic disease complicated by PJP, stratified by time to TMP/SMX initiation

Variables	Total (n = 37)	TMP-SMX ≤ 7 days^1^ (n = 27)	TMP-SMX > 7 days^2^ (n = 10)	P-value
Age (years), mean ± SD	50.1 ± 13.4	52.3 ± 13.2	43.9 ± 12.5	0.105
Sex (male), n (%)	9 (24.3)	7 (25.9)	2 (20.0)	0.999
Underlying rheumatic diseases, n (%)				
Systemic lupus erythematosus	14 (37.8)	9 (33.3)	5 (50.0)	0.454
Dermatomyositis	11 (29.7)	6 (22.2)	5 (50.0)	0.125
Others^3^	12 (32.4)	12 (44.4)	0 (00.0)	NA
Comorbidity, n (%)				
Hypertension	7 (18.9)	4 (14.8)	3 (30.0)	0.360
Diabetes mellitus	4 (10.8)	3 (11.1)	1 (10.0)	0.999
Heart failure	6 (16.2)	4 (14.8)	2 (20.0)	0.653
Chronic renal dysfunction	10 (27)	7 (25.9)	3 (30.0)	0.999
Interstitial Pneumonia	17 (45.9)	10 (37.0)	7 (70.0)	0.136
Laboratory data, n (%) or median (IQR)				
LDH > 500 U/L	21(56.8)	13(48.1)	8(80.0)	0.137
Lymphocyte count (10^9^/L)	0.5 (0.3–1.0)	0.5 (0.3–1.0)	0.6 (0.2–0.8)	0.709
Albumin (g/L)	27.0 (23.4–30.4)	27.0 (23.5–30.0)	29.0 (23.1–30.2)	0.662
CD4 + lymphocyte count (cells/µL)	121.8 (52.0–227.0)	89.9 (59.0–192.0)	153.5 (60.8–243.5)	0.783
PaO_2_/FiO_2_	190.0 (120.0–263.5)	221.0 (145.0–270.0)	125.0 (100.0–222.5)	0.089
C reactive protein (mg/L)	58.7 (22.4–110.1)	75.99 (25.2–113.9)	50.0 (15.0–70.5)	0.183
CMV viremia, n (%)	16 (43.2)	13 (48.2)	3 (30.0)	0.461
Medication before admission				
Cyclophosphamide use, n (%)	9 (24.3)	9 (33.3)	0 (0.0)	NA
≧ 2 immunosuppressive agents use, n (%)	8 (21.6)	7 (25.9)	1 (10.0)	0.404
Methylprednisolone equivalent glucocorticoids (mg/d), median (IQR)	40.0 (28.8–54.0)	40.0 (30.0–50.0)	40.0 (21.5–52.5)	0.857
In-hospital treatment				
Methylprednisolone equivalent glucocorticoids (mg/d), median (IQR)	47.3 (37.7–65.7)	43.2 (36.5–57.1)	72.2 (37.2–86.5)	0.039
Caspofungin use, n (%)	30 (81.1)	20 (74.1)	10 (100.0)	0.155
Mechanical ventilation, n (%)	17 (45.9)	8 (29.6)	9 (90.0)	0.002
90-day PJP-related mortality, n (%)	15 (40.5)	7 (25.9)	8 (80.0)	0.006

^1^TMP-SMX ≤ 7 days: time from initial symptom to TMP/SMX use no more than 7 days; the dosage of TMP/SMX used for treatment of PJP is 15–20 mg/kg TMP and 75–100 mg/kg SMX per day

^2^TMP-SMX > 7 days: time from initial symptom to TMP/SMX use more than seven days; the dosage of TMP/SMX used for treatment of PJP is 15–20 mg/kg TMP and 75–100 mg/kg SMX per day

^3^Others: including Sjogren’s syndrome (pSS), Adult-onset Still’s disease (AoSD), Anti-synthetase syndrome (ASS), Rheumatoid Arthritis (RA), Arteritis

*PJP* *Pneumocystis jirovecii* pneumonia, *TMP/SMX* trimethoprim-sulfamethoxazole, *IQR* inter-quartile range, *LDH* lactate dehydrogenase, *NA* not applicable

A comparison of clinical baseline data in deceased and surviving patients was presented in Table 2. There were no differences in age, sex, basic rheumatic diseases, comorbidity diseases, previous immunosuppressive agents use, daily exposure dose of glucocorticoid within three months between death and survival group. However, we found higher PaO2/FiO2 ratios (246.5 vs. 120.0, P < 0.001) and serum albumin levels (28.2 g/L vs. 23.8 g/L, P = 0.034), fewer high level of LDH (36.4% vs. 86.7%, P = 0.003) and early treatment with TMP/SMX (9.1% vs. 53.3%, P = 0.006) in survivors compared to non-survivors. Besides, survivors required a lower dose of glucocorticoids (41.3 mg/d vs. 73.3 mg/d, P < 0.001) and less mechanical ventilation (13.6% vs. 93.3%, P < 0.001) compared to those non-survivors.

**Table 2:** Clinical characteristics and treatment of patients with rheumatic disease complicated by PJP, stratified by 90-day mortality

Variables	Survival (n = 22)	Death (n = 15)	P-value
Age (years), mean ± SD	50.0 ± 13.3	50.1 ± 14.1	0.977
Sex (male), n (%)	5 (22.7)	4 (26.7)	0.784
Underlying rheumatic diseases, n (%)			
Systemic lupus erythematosus	8 (36.4)	6 (40.0)	0.999
Dermatomyositis	6 (27.3)	5 (33.3)	0.728
Others	8 (36.4)	4 (26.7)	0.724
Comorbidity, n (%)			
Hypertension	4 (18.2)	3 (20.0)	0.890
Diabetes mellitus	2 (9.1)	2 (13.3)	0.683
Heart failure	3 (13.6)	3 (20.0)	0.606
Chronic renal dysfunction	5 (22.7)	5 (33.3)	0.476
Interstitial pneumonia	8 (36.4)	9 (60.0)	0.157
Laboratory, n (%) or median (IQR)			
LDH > 500 U/L	8 (36.4)	13 (86.7)	0.003
Lymphocyte count (10^9^/L)	0.6 (0.30–1.0)	0.4 (0.2–0.8)	0.345
CD4 + lymphocyte count (cells/µL)	137.9 (65.6–207.8)	82.0 (41.5–213.0)	0.404
Albumin (g/L)	28.2 (26.0–31.5)	23.8 (21.4–29.6)	0.034
PaO_2_/FiO_2_	246.5 (192.5–280.0)	120.0 (105.0–147.5)	< 0.001
C reactive protein (mg/L)	49.9 (23.7–92.8)	90.7 (36.1–117.9)	0.337
CMV viremia, n (%)	8 (36.4)	8 (53.3)	0.306
Medication before admission			
Cyclophosphamide use, n (%)	9 (40.9)	0 (0.0)	NA
≧ 2 immunosuppressive agents use, n (%)	5 (22.7)	3 (20.0)	0.843
Methylprednisolone equivalent glucocorticoids (mg/d), median (IQR)	40.0 (30.0–50.0)	40.0 (24.8–60.0)	0.596
In-hospital treatment			
TMP-SMX > 7 days, n (%)	2 (9.1)	8 (53.3)	0.006
Methylprednisolone equivalent glucocorticoids (mg/d), median (IQR)	41.3 (32.2–52.1)	73.3 (50.7–85.2)	< 0.001
Caspofungin use, n (%)	16 (72.7)	14 (93.3)	0.116
Mechanical ventilation, n (%)	3 (13.6)	14 (93.3)	< 0.001

*PJP* *Pneumocystis jirovecii* pneumonia, *TMP/SMX* trimethoprim-sulfamethoxazole, *IQR* inter-quartile range, *LDH* lactate dehydrogenase, *NA* not applicable

Univariate and multivariate analyses of risk factors for mortality are illustrated in Table 3. Multivariate cox analyses showed that patients who were prescribed TMP/SMX after 7 days (HR: 5.9; 95% CI: 1.14–30.43; P = 0.034) and a higher level of LDH (HR: 6.0; 95% CI: 1.13–31.78; P = 0.035) were associated with higher mortality in patients with rheumatic disease complicated by PJP.

**Table 3:** Univariate and multivariate analysis of risk factors for 90-day PJP-related mortality

Variable	Univariable		Multivariable	
	HR (95% CI)	P-value	HR (95%CI)	P-value
TMP-SMX > 7 days	4.0 (1.4, 11.3)	0.009	5.9 (1.1, 30.4)	0.034
Age, per 1 years old	1.0 (1.0, 1.0)	0.827		
Sex (female or male)	1.2 (0.4, 3.8)	0.757		
Interstitial Pneumonia (yes or no)	1.9 (0.7, 5.5)	0.210		
LDH > 500 U/L	7.2 (1.6, 32.1)	0.010	6.0 (1.1, 31.8)	0.035
Lymphopenia < 1000 cells/µL	0.8 (0.2, 2.7)	0.660		
CD4 + lymphocyte count < 200 cells/µL	1.0 (0.3, 3.0)	0.947		
Albumin < 30 g/L	1.7 (0.5, 6.2)	0.393		
Caspofungin (yes or no)	3.7 (0.5, 28.0)	0.210		
Methylprednisolone equivalent glucocorticoids (mg/d), per 10 mg/d	1.9 (0.5, 6.7)	0.353		

*PJP* *Pneumocystis jirovecii* pneumonia, *HR* hazard ratio, *CI* confidence interval, *TMP/SMX* trimethoprim-sulfamethoxazole, *LDH* lactate dehydrogenase

**Fig. 1:**
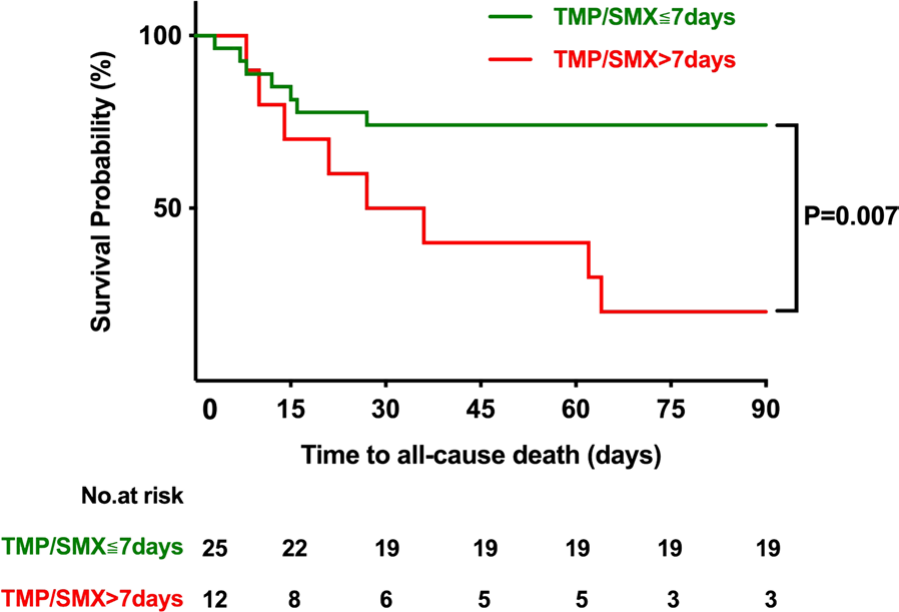
Kaplan–Meire survival plot for 90 days follow-up to PJP-related mortality

Kaplan–Meier survival analysis in Fig. 1 shows that patients who received early treatment with TMP/SMX within 7 days had a better prognosis than those who did not receive TMP/SMX within 7 days (P = 0.007).

## Discussion

This study reports that patients with rheumatic disease complicated by PJP who received TMP/SMX > 7 days after initial symptoms had an increased risk of 90-day mortality compared with those who received TMP/SMX within 7 days. Additionally, a higher level of LDH also correlated with a worse prognosis in patients with rheumatic diseases complicated by PJP.

Previous studies have shown that long-term exposure to glucocorticoids and immunosuppressant therapy increases the risk of PJP infection in patients with rheumatic diseases [16]. Due to the immunosuppressive condition of patients, they are prone to develop opportunistic infections, which may be exacerbated death [16–18]. Similar to previous studies [13], in our research, the 90-day mortality rate of PJP patients was as high as 40.5%. Current guidelines recommended PJP prophylaxis for patients receiving immunosuppressive drugs, including glucocorticoids [19]. Besides, there are several researches demonstrated that TMP-SMX prophylaxis significantly reduces the PJP incidence with a favorable safety profile in patients with rheumatic disease receiving prolonged, high-dose glucocorticoids[11, 12, 15, 19].

The characteristic presentation of PJP included acute fever, dyspnea, nonproductive cough, hypoxemia, and even thoracic pain in clinical settings [14]. PJP presented as bilateral interstitial infiltrates and bilateral ground-glass attenuation on thoracic CT scans [20]. PJP should be highly suspected when immunocompromised patients with these typical clinical symptoms and CT signs [21], and prompt antimicrobial treatment against *P. jirovecii* should be triggered [7, 22, 23]. The metagenomic next-generation sequencing (NGS) field has gradually developed and matured in recent years. NGS technology has the advantages of being unaffected by the use of antibiotics and having a short detection time. With the promotion of NGS application in clinical settings, the detection rate of *P. jirovecii* is significantly increasing [24, 25]. In present study, PJP was identified by NGS of BALF in 25 of the 37 patients, and by NGS of blood in 11 patients, and one patients was diagnosed by conventional microscopic staining of sputum specimens.

Since delayed treatment could increase mortality and the need for mechanical ventilation, immediate initiation of PJP-specific therapy is critical [7, 22, 23]. Based on the real-world clinical setting, for patients suspected of PJP, first week was considered the acceptable cut-off point for TMP/SMX treatment, so we divided the patients into two groups to be compared. The baseline characteristics of patients in the two groups were comparable; however, the outcomes differed even after multivariate analyses. Our research suggests that early administration of TMP/SMX within the first week of symptoms was associated with better clinical outcome. Cox regression analysis showed that initiation of TMP/SMX after > 7 days from the onset of clinical symptoms was related to higher 90-day PJP-related mortality than those who received TMP/SMX treatment within 7 days. Our study reconfirms that early treatment with TMP/SMX within first week is a critical measure to reduce mortality among patients with rheumatic disease complicated with PJP.

Interstitial lung disease (ILD) may be associated with increased pulmonary colonization by *Pneumocystis jirovecii* [26], and there is an increased risk of PJP in patients with autoimmune rheumatic diseases[27]. In present study, a higher proportion of patients with interstitial lung disease were in the delayed treatment group (70% vs 37%, P = 0.136). Considering similar radiologic manifestation, the diagnosis of PJP might be more difficult in patients with ILD. In the rheumatic patients combined with interstitial pneumonia, there were more patients treated with TMP/SMX early in survivors compared to non-survivors, however, it was only borderline statistically significant (87.5% vs. 33.3%, P = 0.05).

Previous clinical studies have shown that high LDH levels were a risk factor for adverse outcomes in PJP patients [28]. A clinical retrospective study over 17 years found that initial LDH levels can be used as a stratification tool to identify patients with a high risk of mortality [29]. In addition, the prolonged usage of glucocorticoids, delayed anti-*P. jirovecii* therapy, hypoalbuminemia, CMV infection, acute respiratory distress syndrome, severe pulmonary edema, respiratory failure, the need for mechanical ventilation, and high-dose glucocorticoid therapy during hospitalization were all predictive of poor outcomes [21, 30–35].

This study demonstrated that a higher level of LDH and lower PaO2/FiO2 ratio in the early stage of PJP resulted in adverse outcomes. Consistent with previous studies, the multivariate analyses showed that higher level LDH was associated with a worse prognosis in PJP patients with rheumatic diseases.

The study has several limitations. First, data loss is unavoidable due to the retrospective experimental design, such as the loss of BALF cell counts, which leads to the loss of some indicators of PJP prognoses. Second, the study's sample size was limited due to the disease’s rarity. Thirdly, our single-center retrospective design reduced the generalizability of our findings. In the future, we will undertake high-quality multi-center studies to analyze the risk factors affecting the prognosis of PJP patients with rheumatic diseases.

## Conclusion

Patients with rheumatic disease complicated by PJP are associated with poor prognoses regardless of specific anti-*P. jirovecii* treatment. Time to TMP/SMX initiation > 7 days after initial symptoms and higher serum LDH levels were associated with adverse outcomes in patients with rheumatic disease with PJP.

## Data Availability

The datasets used and/or analyzed during the current study are available from the corresponding author on reasonable request.

## Abbreviations

BALF: Bronchoalveolar lavage fluid
CI: Confidence interval
CT: Computed tomography
CMV: Cytomegalovirus
HIV: Human immunodeficiency virus
HR: Hazard ratio
IM: Inflammatory myopathy
LDH: Lactate dehydrogenase
NGS: Next-generation sequencing
PJP: *Pneumocystis jiroveci* pneumonia
SLE: Systemic lupus erythematosus
TMP/SMX: Trimethoprim/sulfamethoxazole
